# NR1D1 controls skeletal muscle calcium homeostasis through myoregulin repression

**DOI:** 10.1172/jci.insight.153584

**Published:** 2022-09-08

**Authors:** Alexis Boulinguiez, Christian Duhem, Alicia Mayeuf-Louchart, Benoit Pourcet, Yasmine Sebti, Kateryna Kondratska, Valérie Montel, Stéphane Delhaye, Quentin Thorel, Justine Beauchamp, Aurore Hebras, Marion Gimenez, Marie Couvelaere, Mathilde Zecchin, Lise Ferri, Natalia Prevarskaya, Anne Forand, Christel Gentil, Jessica Ohana, France Piétri-Rouxel, Bruno Bastide, Bart Staels, Helene Duez, Steve Lancel

**Affiliations:** 1 Université de Lille, Inserm, CHU Lille, Institut Pasteur de Lille, U1011-EGID, Lille, France.; 2 Université de Lille, Inserm, U1003 — PHYCEL — Physiologie Cellulaire, Lille, France.; 3 Université de Lille, Université d’Artois, Université du Littoral Côte d’Opale, EA 7369 — URePSSS — Unité de Recherche Pluridisciplinaire Sport Santé Société, Lille, France.; 4 Inovarion, Paris, France.; 5 Sorbonne Université-UMRS974-Inserm-Institut de Myologie, Paris, France.; 6 MyoLine Immortalization Platform, Sorbonne Université-UMRS974-Inserm-Institut de Myologie, Paris, France.

**Keywords:** Cell Biology, Muscle Biology, Calcium, Muscle

## Abstract

The sarcoplasmic reticulum (SR) plays an important role in calcium homeostasis. SR calcium mishandling is described in pathological conditions, such as myopathies. Here, we investigated whether the nuclear receptor subfamily 1 group D member (NR1D1, also called REV-ERBα) regulates skeletal muscle SR calcium homeostasis. Our data demonstrate that NR1D1 deficiency in mice impaired sarco/endoplasmic reticulum calcium ATPase–dependent (SERCA-dependent) SR calcium uptake. NR1D1 acts on calcium homeostasis by repressing the SERCA inhibitor myoregulin through direct binding to its promoter. Restoration of myoregulin counteracted the effects of NR1D1 overexpression on SR calcium content. Interestingly, myoblasts from patients with Duchenne muscular dystrophy displayed lower NR1D1 expression, whereas pharmacological NR1D1 activation ameliorated SR calcium homeostasis and improved muscle structure and function in dystrophic *mdx/Utr^+/–^* mice. Our findings demonstrate that NR1D1 regulates muscle SR calcium homeostasis, pointing to its therapeutic potential for mitigating myopathy.

## Graphical abstract

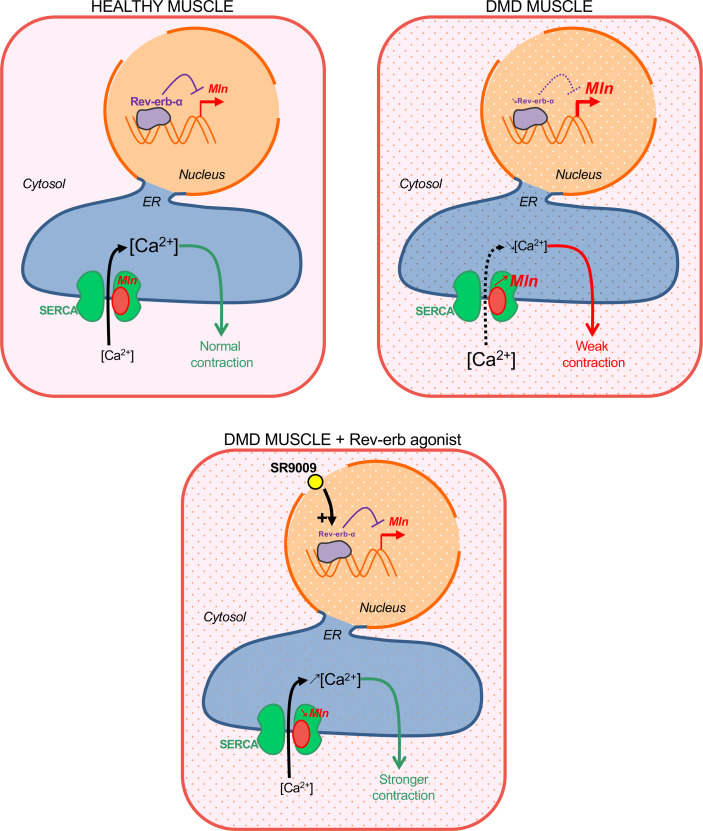

## Introduction

Skeletal muscle is not only required for movements, but it is also crucial for other vital functions, such as respiration. Myopathies (1, 2), among which Duchenne muscular dystrophy (DMD) is one of the most prevalent forms, result in progressive muscle weakness and wasting and can lead to premature death. Despite progress in gene therapy, DMD still remains an unmet medical need; new strategies are needed to alleviate skeletal muscle degeneration.

Calcium (Ca^2+^) is important for muscle contractile function, and its subcellular distribution is tightly regulated by several pumps and channels (3). Ca^2+^ is stored in the endoplasmic/sarcoplasmic reticulum (ER/SR), where it mainly interacts with Ca^2+^-binding proteins such as calsequestrin (4). Following an action potential, membrane depolarization triggers a massive Ca^2+^ release through the ryanodine receptor (RyR), hence promoting contraction and muscle force generation. Ca^2+^ reuptake from the cytosol into the SR lumen by the sarco/endoplasmic reticulum calcium ATPase (SERCA) allows muscle relaxation and a new cycle of contraction/relaxation. Because SERCA Ca^2+^ pump activity plays a prominent role in skeletal muscle contractility, it is tightly regulated by different factors, including the recently discovered inhibitory SR transmembrane α helix micropeptide myoregulin (MLN) (5). Disturbances of these fine-tuned processes have been observed in DMD, where chronically elevated cytosolic Ca^2+^ concentrations (6, 7), decreased SERCA activity (8–10), and reduced Ca^2+^ release upon excitation can be observed (2, 9, 11). Progressive loss of muscle force generation, as observed in the *mdx* mouse model of DMD, is explained by the absence of homeostatic return to basal cytosolic Ca^2+^ levels between two contractions (12), underlying the importance of normal SERCA activity for muscle function.

We have previously reported that the druggable nuclear receptor subfamily 1 group D member (NR1D1), a transcriptional repressor also known as REV-ERBα (13), improves skeletal muscle function and exercise capacity (14). NR1D1 improves mitochondrial function along with increased mitochondrial biogenesis (14). Here, we investigated whether NR1D1 controls additional mechanisms accounting for skeletal homeostasis. We particularly assessed whether NR1D1 modulates the major SR function, i.e., Ca^2+^ handling. We demonstrate that NR1D1, through its transcriptional repressive activity on the *Mln* gene, increased SERCA activity and SR Ca^2+^ content in mouse and human muscle cells. Importantly, pharmacological NR1D1 activation with SR9009 decreased *Mln* expression, improved calcium handling, enhanced force generation, and minimized tissue damage in severely dystrophic *mdx/utr^+/–^* mice. Overall, we believe that our results identify NR1D1 as a new regulator of SR calcium homeostasis that may represent a therapeutic target in skeletal muscle disorders related to impaired reticular calcium homeostasis, such as myopathies.

## Results

### NR1D1 improves muscle force along with SR Ca^2+^ homeostasis.

**Figure 1:**
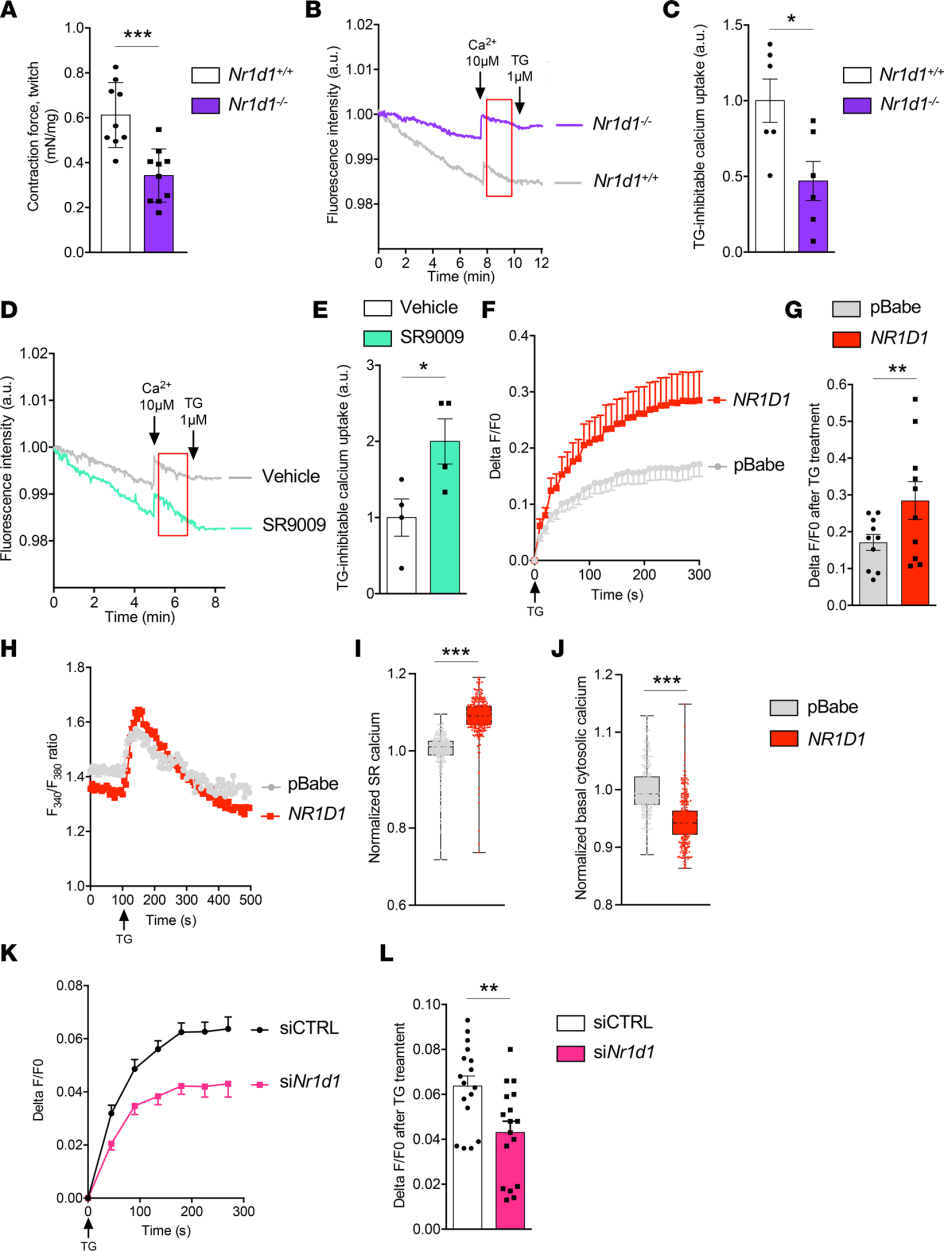
NR1D1 regulates SR Ca^2+^ homeostasis in skeletal muscle. (**A**) In situ measurement of gastrocnemius-developed force upon an electrical stimulus in wild-type (*Nr1d1^+/+^*) and *Nr1d1^–/–^* mice. ***P = 0.0004 vs. *Nr1d1^+/+^*, unpaired *t* test; *n* = 9–10. (**B**) Representative curves of SERCA-inhibitable Ca^2+^ uptake in microsomal fractions prepared from muscle from *Nr1d1^+/+^* and *Nr1d1^–/–^* mice. Decrease in fluorescence indicates Ca^2+^ uptake by microsomes. Arrows indicate Ca^2+^ or thapsigargin (TG) injections. The red rectangle was used for (**C**) slope calculation of fluorescence decrease, indicative of specific SERCA Ca^2+^ uptake. *P = 0.0203 vs. *Nr1d1^+/+^*, unpaired *t* test; *n* = 6. (**D**) Representative curves of SERCA-inhibitable Ca^2+^ uptake in microsomes prepared from muscle from vehicle- or SR9009-treated wild-type mice. (**E**) Slopes of the decreasing fluorescence over time. *P = 0.0408 vs. vehicle, unpaired *t* test; *n* = 4. (**F**) TG-induced SR Ca^2+^ release in control pBabe- and *NR1D1*-overexpressing C2C12 myotubes. Cells were loaded with Fluo4-AM to detect cytosolic Ca^2+^. SR Ca^2+^ content depletion was induced by TG (1 μM) (*n* = 10). (**G**) Delta F/F0 ratio normalized to pBabe values, obtained 5 minutes after TG-induced Ca^2+^ release. **P = 0.007 vs. pBabe, unpaired *t* test; *n* = 10. (**H**) Representative Fura-2/AM experiments (ratio F340/F380) in pBabe- and *NR1D1*-overexpressing cells. (**I**) Normalized SR calcium concentration released upon TG treatment in pBabe- and *NR1D1*-overexpressing cells. (**J**) Normalized basal cytosolic calcium concentration (mean of the 100 first seconds) in pBabe- and *NR1D1*-overexpressing cells. (**I** and **J**) Box-and-whisker plots show all points, with minimums and maximums (*n* > 300 in each group). ***P < 0.0001 vs. pBabe, unpaired *t* test. (**K**) TG-induced SR Ca^2+^ release in C2C12 cells transfected by control (siCTRL) or *Nr1d1* siRNA (siNr1d1). Results are shown as Delta F/F0 ratio (*n* = 17). (**L**) Delta F/F0 ratio normalized to siCTRL values obtained 5 minutes after TG-induced Ca^2+^ release. **P = 0.0044 vs. siCTRL, unpaired *t* test; *n* = 17. Data are shown as the mean ± SEM.

We first aimed to determine whether NR1D1 is important for muscle force generation and found that muscle contraction was reduced by approximately 50% in *Nr1d1^–/–^* mice compared with their wild-type (*Nr1d1^+/+^*) littermate controls (Figure 1A and Supplemental Figure 1A; supplemental material available online with this article; https://doi.org/10.1172/jci.insight.153584DS1). Because Ca^2+^ homeostasis is essential for muscle force generation, we next determined whether NR1D1 controls SR Ca^2+^ handling. Muscle microsomes, i.e., sarcoplasmic vesicles, were prepared from *Nr1d1^–/–^* and *Nr1d1^+/+^* littermates. SERCA-dependent SR Ca^2+^ uptake capacity was measured over time after the addition of Ca^2+^ pulses or thapsigargin (TG), a potent inhibitor of SERCA activity (15), by using a fluorescent probe detecting extramicrosomal Ca^2+^. The slope of fluorescence decrease, i.e., SR Ca^2+^ uptake, was significantly lower in *Nr1d1^–/–^* mice compared with that in *Nr1d1^+/+^* mice, revealing a reduction in SERCA activity in absence of NR1D1 (Figure 1, B and C). By contrast, in vivo treatment of wild-type mice with the NR1D1 agonist SR9009 led to improved SERCA activity measured in muscle microsomes prepared from these mice (Figure 1, D and E). In order to measure passive Ca^2+^ release from SR as a surrogate of its initial Ca^2+^ content, differentiated *NR1D1*-overexpressing or control (pBabe) C2C12 myotubes were loaded with the cytosolic Ca^2+^-sensitive probe Fluo4-AM and then challenged with TG to release Ca^2+^ from the SR. TG addition led to a greater elevation in Fluo4 fluorescence in *NR1D1*-overexpressing cells compared with pBabe, revealing that NR1D1 overexpression is associated with increased SR Ca^2+^ content (Figure 1, F and G). Consistently, similar results were obtained using the cytosolic Ca^2+^-sensitive probe Fura-2 AM, which is a dual-excitation, single-emission Ca^2+^ indicator, which avoids possible loading artifacts (Figure 1, H and I). In agreement with these findings, basal cytosolic calcium, buffered at least in part by the SR, was reduced by *NR1D1* overexpression (Figure 1J). In contrast, siRNA-mediated *Nr1d1* silencing (Supplemental Figure 1B) decreased reticular calcium content in C2C12 myotubes (Figure 1, K and L). Together, these data indicate that NR1D1 controls SR Ca^2+^ homeostasis in skeletal muscle.

### NR1D1 controls Ca^2+^ homeostasis through direct repression of myoregulin expression.

**Figure 2:**
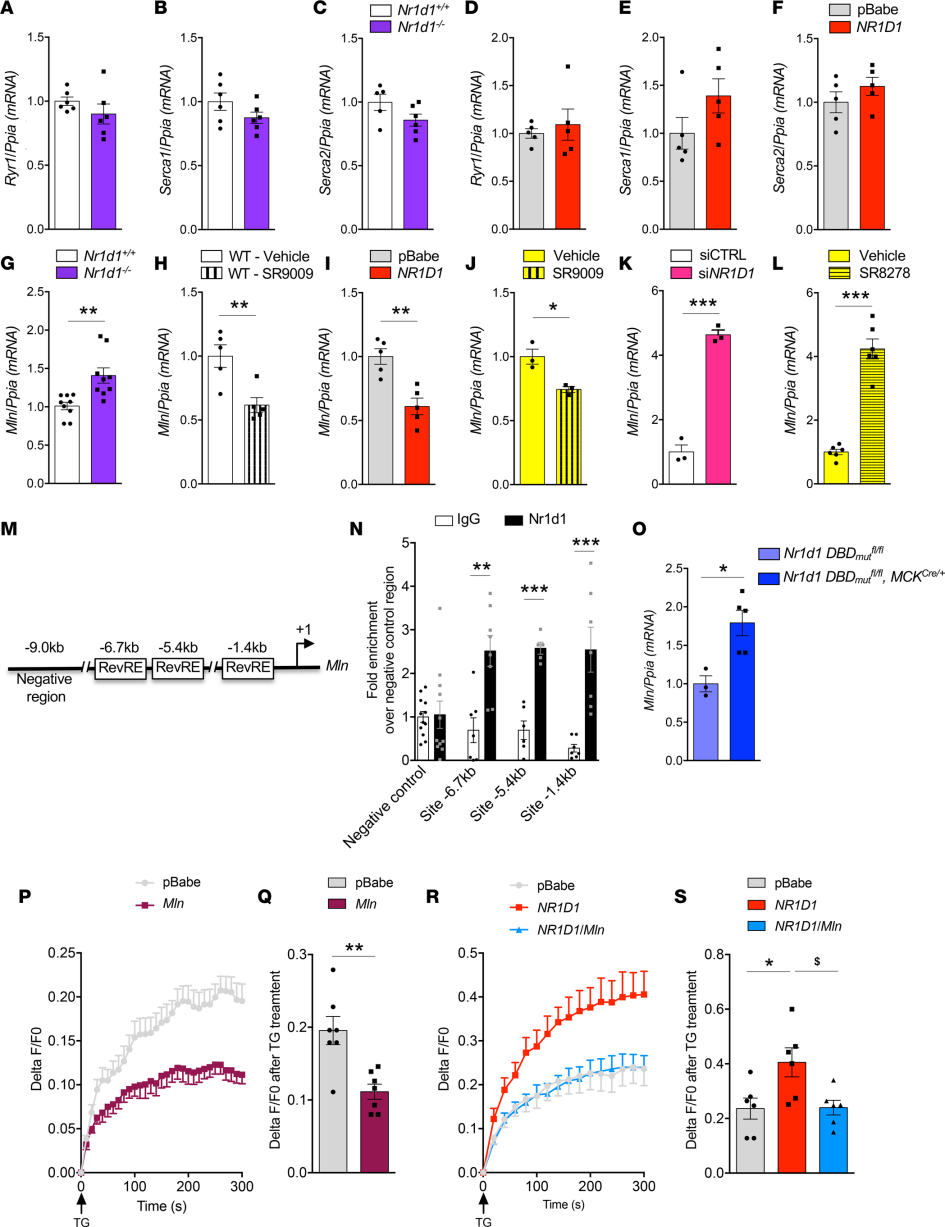
NR1D1 represses myoregulin expression through direct binding to its promoter. (**A–L**) *RyR1*, *Serca1*, and *Serca2* gene expression in (**A–C**) muscle from *Nr1d1^+/+^* and *Nr1d1^–/–^* mice (*n* = 6) and (**D–F**) pBabe- and *NR1D1*-overexpressing differentiated C2C12 (*n* = 3). Myoregulin (*Mln*) expression in (**G**) muscle from *Nr1d1^+/+^* and *Nr1d1^–/–^* mice (**P = 0.0025 vs. *Nr1d1^+/+^; n* = 9), (**H**) muscle from SR9009-treated wild-type animals (**P = 0.0069 vs. vehicle; *n* = 5), (**I**) *NR1D1-*overexpressing C2C12 (**P = 0.0023 vs. pBabe; *n* = 5), (**J**) C2C12 treated with 10 μM of NR1D1 agonist SR9009 (*P = 0.0155 vs. DMSO; *n* = 3), (**K**) C2C12 transfected with si*Nr1d1* (***P < 0.001 vs. siCTRL; *n* = 3), and (**L**) C2C12 treated with 10 μM of NR1D1 antagonist SR8278 (***P < 0.0001 vs. DMSO; *n* = 6). Unpaired *t* test. (**M**) Schematic representation of *Mln* promoter, indicating 3 putative *Rev-erb*α response elements (RevRE), located approximately 1.4 kb, 5.4 kb, and 6.7 kb upstream of the transcription initiation site. (**N**) ChIP analysis using an anti-NR1D1 antibody or control IgG. –6.7 kb, **P = 0.0017; –5.4 kb, ***P < 0.0001; –1.4 kb, ***P = 0.001 vs. IgG; *n* = 6–8 mice. (**O**) *Mln* expression in mice with muscle-specific expression of a mutated isoform of NR1D1 lacking the DNA-binding domain (*Nr1d1 DBD_mut_^fl/fl^, MCK^Cre/+^*) and control *Nr1d1 DBD_mut_^fl/fl^* mice. *P = 0.0139 vs. *Nr1d1 DBD_mut_^fl/fl^*, unpaired *t* test; *n* = 3–5. (**P**) TG-induced SR Ca^2+^ release in pBabe- and *Mln*-overexpressing differentiated C2C12 expressed as Delta F/F0 ratio. *n* = 7. (**Q**) Peak fluorescence intensity of TG-induced SR Ca^2+^ release in pBabe- and *Mln*-overexpressing differentiated C2C12, normalized to pBabe. **P = 0.024 vs. pBabe, unpaired *t* test; *n* = 7. (**R**) TG-induced SR Ca^2+^ release in pBabe- and *NR1D1*- and *NR1D1/Mln*-overexpressing differentiated C2C12 expressed as Delta F/F0 ratio; *n* = 6. (**S**) Peak fluorescence intensity of TG-induced SR Ca^2+^ release in pBabe- and *NR1D1*- and *NR1D1/Mln*-overexpressing differentiated C2C12, normalized to pBabe. *P < 0.026 vs. pBabe, ^$^*P* < 0.0293 vs. NR1D1, 1-way ANOVA, Tukey’s multiple comparison test; *n* = 6. Data are shown as the mean ± SEM.

We then aimed to identify the mechanism by which NR1D1 regulates calcium homeostasis. Because skeletal muscle SR Ca^2+^ homeostasis is mainly controlled by the ryanodine receptor RyR1, SERCA1, and SERCA2, we determined whether NR1D1 controls their expression. Whereas *Ryr1*, *Serca1*, and *Serca2* mRNA expression was identical in *Nr1d1^+/+^* and *Nr1d1^–/–^* mice (Figure 2, A–C), as well as in pBabe- and *NR1D1*-overexpressing cells (Figure 2, D–F), expression of *Mln*, a recently identified skeletal muscle–specific SERCA inhibitor (5), was significantly higher in skeletal muscle from *Nr1d1^–/–^* mice compared with that in control littermates (Figure 2G). Treatment with SR9009 in vivo to activate NR1D1 significantly decreased *Mln* expression (Figure 2H). Consistently, *NR1D1* overexpression or NR1D1 pharmacological activation with SR9009 decreased *Mln* expression in C2C12 cells (Figure 2, I and J), whereas *NR1D1*-targeting siRNA or cell treatment with the NR1D1 antagonist SR8278 induced *Mln* expression (Figure 2, K and L).

In silico analysis identified at least 3 putative Rev-erb response elements (RevREs) located 1.4, 5.4, and 6.7 kb upstream of the *Mln* transcription start site (Figure 2M). Using ChIP–quantitative PCR (qPCR) experiments performed on mouse skeletal muscle, we demonstrate that NR1D1 bound to these 3 regions (Figure 2N). To test whether direct NR1D1 binding to the *Mln* gene is required for its regulation, we used skeletal muscle–specific mutant mice expressing a DNA-binding domain–deficient (DBD-deficient) NR1D1 protein. As observed in *Nr1d1^–/–^* mice, *Mln* expression was higher in skeletal muscle–specific mutant mice compared with wild-type floxed littermates (Figure 2O). Altogether, these data reveal that NR1D1 represses *Mln* gene expression by direct binding to its promoter.

To functionally demonstrate that MLN is key in the regulation of muscle Ca^2+^ homeostasis by NR1D1, we aimed to restore MLN expression in NR1D1-overexpressing cells. As expected, overexpression of *MLN* alone, by viral vector transduction in C2C12 myotubes, reduced SR Ca^2+^ stores compared with that in control pBabe cells (Figure 2, P and Q). More importantly, *MLN* overexpression in *NR1D1*-overexpressing cells normalized Ca^2+^ handling (Figure 2, R and S).

### Pharmacological NR1D1 activation improves SERCA-dependent Ca^2+^ uptake and alleviates the dystrophic phenotype in DMD.

**Figure 3:**
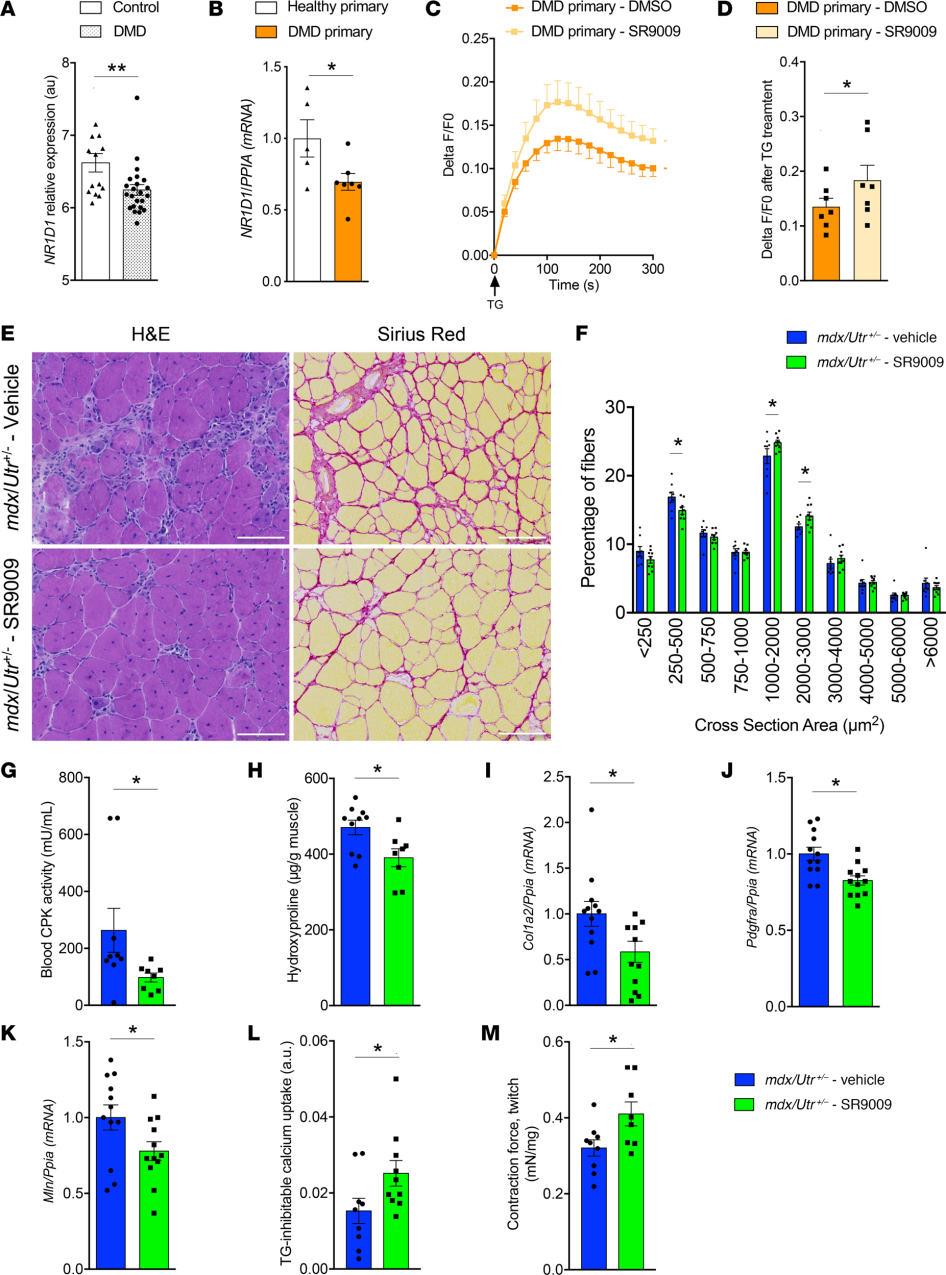
NR1D1 activation alleviates Duchenne muscular dystrophy features both in mice and human myoblasts. (**A**) *NR1D1* expression in muscle biopsies from controls (*n* = 14) and patients with Duchenne muscular dystrophy (DMD, *n* = 23). Data are from GEO data set GSE6011. **P = 0.0092, unpaired *t* test. (**B**) *NR1D1* expression in control or DMD primary myotubes. *P = 0.0404 vs. control cells, unpaired *t* test; *n* = 5–7. (**C**) Representative curves and (**D**) peak fluorescence intensity of thapsigargin-induced (TG-induced) sarcoplasmic reticulum (SR) Ca^2+^ release in myoblasts from controls or patients with DMD treated with SR9009 (10 μM) or vehicle. Cells were loaded with Fluo4-AM and SR Ca^2+^ release was induced by the addition of 1 μM TG. Results are expressed as (mean ± SEM) the Delta F/F0 ratio. **P = 0.0049 vs. control cells, unpaired *t* test; *n* = 3 controls, *n* = 7 in both DMD groups. (**E**) H&E and Sirius red staining of tibialis anterior muscles obtained from vehicle- and SR9009-injected *mdx/Utr^+/–^* mice. Scale bars: 100 μm. (**F**) Myofiber cross-sectional area distribution. *P < 0.05 vs. vehicle-treated *mdx/Utr^+/–^* mice by 2-way ANOVA; *n* = 7–9. (**G**) Circulating creatine phosphokinase (CPK) activity. *P = 0.0329 vs. vehicle-treated *mdx/Utr^+/–^* animals; *n* = 8–9. (**H**) Muscular hydroxyproline. *P = 0.0172; *n* = 8–10. (**I**) *Col1a2* (*P = 0.0314), (**J**) *Pdgfra* (*P = 0.0164), and (**K**) *Mln* (*P = 0.0402) gene expression; *n* = 8–12. (**L**) SERCA activity (*n* = 8–10) in muscular microsomes from *mdx/Utr^+/–^* mice treated for 20 days with SR9009 (100 mg/kg) or vehicle. *P = 0.0266. (**M**) In situ measurement of gastrocnemius-developed force. *P = 0.0301; *n* = 4–9. (**G**–**M**) Unpaired *t* test.

Calcium homeostasis is known to be impaired in several myopathies (2, 16). To determine whether this could be due, at least in part, to a deregulation of NR1D1 and its downstream targets, we first analyzed its expression using publicly available microarray data sets from dystrophic muscles. Interestingly, we found that *NR1D1* mRNA was expressed, albeit to significantly lower levels, in patients with DMD from different studies (17–19) (Figure 3A and Supplemental Figure 2, A and B). The same was observed in muscle biopsies from patients with DMD (Supplemental Figure 2C). Likewise, *NR1D1* expression was reduced by approximately 30% in DMD muscle cells compared with human control cells (Figure 3B). These data indicate altered NR1D1 expression, and hence action, may be associated with Duchenne dystrophy, pointing to NR1D1 as an interesting target in this myopathy specifically. Therefore, we tested whether pharmacological NR1D1 activation by SR9009 may improve Ca^2+^ homeostasis in primary cells from patients with DMD. A significantly higher SR Ca^2+^ release triggered by TG was measured in SR9009-treated compared with vehicle-treated DMD cells (Figure 3, C and D).

To determine whether this was related to MLN, we used immortalized DMD myotubes because they shared reduced *NR1D1* expression and SR calcium content, as observed in primary cells (Supplemental Figure 2, D–H). Whereas *MLN* overexpression in healthy myotubes reduced SR calcium content (Supplemental Figure 2, I and J), shRNA against *MLN* improved SR calcium content in DMD immortalized myotubes (Supplemental Figure 2, K and L).

To further assess whether pharmacological NR1D1 activation could improve muscle function in a pathological DMD model in vivo, *mdx/Utr^+/–^* mice, which closely recapitulate the features of the human disease, were daily injected with SR9009 for 20 days. Histological analysis revealed that tissue architecture was improved in SR9009-treated mice (Figure 3E and Supplemental Figure 3A), along with a mild, but significant, decrease in small fibers and an increase in medium/large fibers compared with vehicle-injected *mdx*/*Utr*^+/–^ mice (Figure 3F). Circulating blood creatine phosphokinase, which is a marker of muscle damage, was strongly decreased in the SR9009-treated group compared with vehicle-treated *mdx*/*Utr*^+/–^ mice (Figure 3G). Several fibrosis markers, including Sirius red staining (Figure 3E), muscle hydroxyproline quantity (Figure 3H), and *Col1a2* (Figure 3I) and *Pdgfra* (Figure 3J) expression, were also reduced by SR9009 treatment. Next, we evaluated whether SR9009 treatment was able to improve muscle calcium homeostasis and function in this model of myopathy. As expected, based on the data from the genetic models of deletion or overexpression of NR1D1 specifically in skeletal muscle, daily injection of SR9009 for 20 days reduced *Mln* expression in muscle from *mdx/Utr^+/–^* mice (Figure 3K), whereas SERCA activity was improved (Figure 3L); both of these findings were strongly correlated with one another (Supplemental Figure 3B). More importantly, gastrocnemius muscle–developed contraction force was significantly ameliorated by SR9009 treatment in *mdx/Utr^+/–^* mice compared with vehicle-injected littermates (Figure 3M and Supplemental Figure 3C).

In conclusion, a 20-day NR1D1 agonist treatment improved SR homeostasis and muscle function in a mouse model of DMD.

## Discussion

Our data demonstrate that NR1D1 improved calcium homeostasis in skeletal muscle by directly controlling *Mln* expression, and hence SERCA activity. We also report that NR1D1 pharmacological activation by synthetic ligands may reveal therapeutic interests, because it improved calcium homeostasis in cells from patients with DMD and alleviated the myopathy phenotype in *mdx*/*Utr^+/–^* mice.

RyR1 and SERCA1 are the 2 major SR proteins controlling Ca^2+^ fluxes in skeletal muscle. Nevertheless, neither RyR1 nor SERCA1 expression was affected by NR1D1. Therefore, we focused on MLN, the main glycolytic/mixed muscle endogenous SERCA inhibitor (20). MLN is a recently discovered 46–amino acid micropeptide that forms a single transmembrane α helix and interacts with the skeletal muscle SERCA1 isoform to inhibit its pumping activity, thereby decreasing SR Ca^2+^ content (5). Here, we identified NR1D1 as a potentially new direct transcriptional repressor of *Mln* gene expression. Indeed, we demonstrated that NR1D1 binds to 3 RevREs located in the *Mln* promoter via a functional DNA-binding domain. These data are potentially limited: because the anti-NR1D1 antibody used in our ChIP assay was ChIP grade, not ChIP-Seq grade, nonspecific binding to RevREs in the *Mln* promoter remains possible. By modulating Ca^2+^ handling, MLN was proposed to modulate skeletal muscle contractile activity and to represent a promising drug target for improving Ca^2+^-related skeletal muscle disorders and muscle performance (5). Consistently, *Mln* deletion in mice improves skeletal muscle performance (5). Yet, modulators of *Mln* expression remained to be identified. Interestingly, pharmacological NR1D1 activation, which we have shown in previous studies to improve muscle performance in nonpathological contexts (14) and to block glucocorticoid-induced muscle wasting (21), is able to repress *Mln* expression. We were also able to strongly correlate *Mln* expression and calcium uptake activity, despite the absence of validation at the protein level due to the lack of specific anti-MLN commercially available antibodies. Therefore, we bring insights into the molecular mechanisms by which NR1D1 exerts beneficial effects on muscle function and uncover a pathway to control *Mln*, and hence skeletal muscle Ca^2+^ handling and likely contractile function.

We have demonstrated that *NR1D1* is expressed in DMD cells, albeit to a lower extent compared with control human myotubes, suggesting that increasing NR1D1 activity could represent a new therapeutic option in myopathies. In muscle from patients with DMD, the absence of dystrophin causes muscular contraction impairment with altered Ca^2+^ handling, i.e., raised cytosolic Ca^2+^ concentrations and depletion of SR Ca^2+^ stores due to impaired uptake capacity (2, 22). Here, we confirm these data, and we further demonstrate that pharmacological activation of NR1D1 by a synthetic ligand improved SR Ca^2+^ content in human DMD myotubes. This was also observed in vivo in a DMD mouse model in which pharmacological activation of NR1D1 significantly improved muscle histology and reduced damage markers and fibrosis. By itself, and consistent with results from other studies showing that improving Ca^2+^ homeostasis mitigates DMD (7, 22), we can hypothesize that pharmacological NR1D1 activation reduces MLN expression, which may partly contribute to the improved muscle contractility observed in myopathic mice. Yet, we cannot rule out that NR1D1 activation exerts effects on endothelial cells, interstitial mesenchymal cells, immune cells, or satellite cells, contributing to the overall improved phenotype.

Others have reported that NR1D1 antagonism with SR8278 may also improve muscle function, reduce fibrosis, and increase mitochondrial biogenesis in *mdx* mice (23). That study is in apparent contradiction with the present results and with results that, we and authors of ref. 23, have previously published demonstrating that NR1D1 agonism with SR9009 improves muscle mitochondrial function and exercise capacity in healthy mice (14). In addition, we and others have reported that NR1D1 positively controls skeletal muscle mass by counteracting both autophagy (14) and proteasomal-associated fiber atrophy (21) and by promoting myoblast differentiation through mTORC1 signaling pathway activation (24), again supporting a positive action of NR1D1 in skeletal muscle. Although out of scope of this study, these cellular processes may also contribute to the improvement of the myopathy phenotype upon NR1D1 activation. Indeed, we also observed that *Nr1d1*^–/–^ mice displayed impaired tetanic contraction, suggesting that calcium uptake impairment is not the only parameter explaining this muscular deficit. Compensatory mechanisms may interfere, as both Nr1d2 overexpression and knockdown were reported to lead to a similar increase in mitochondrial biogenesis (25). Moreover, SR9009 as well as SR8278 target both NR1D1 and NR1D2 (26) and may also exert NR1D1-independent activities (27). In this study, we have used genetic models of deletion or overexpression of NR1D1 specifically in skeletal muscle and in mouse and human myotubes to support our model and validate the role of NR1D1 in ameliorating muscle calcium handling and improving dystrophy. While this is possibly one reason for the apparent discrepancy between our results and others (23), it should also be noted that we used a different mouse model of muscle dystrophy. While the *mdx* mouse is widely used, it is a very mild model of disease, which is far from the human DMD phenotype (28). In contrast, we used the *mdx*/*Utr^+/–^* model, which presents a profound phenotype more relevant to the human situation, which may also explain why different results were obtained. We also showed, that this pertains to human muscle biopsies (17–19) — although conclusions have to be taken cautiously due to patient age, stage, and treatment heterogeneity — and to myotubes from patients with DMD. Moreover, although it would be impossible to disentangle the two pathways, the beneficial effects of NR1D1 on muscle function could also be related to the combination of two mechanisms. Indeed, by improving mitochondrial function (14), NR1D1 activation could lead to higher ATP availability for calcium pumps and myofibrillar proteins. Even though, for the above-mentioned reasons, the current ligands cannot be used in the clinic, our results suggest further development of more selective NR1D1-activating drugs, which could be of interest in the treatment of myopathies and likely other muscle disorders characterized by altered Ca^2+^ homeostasis.

## Methods

### Study design

Genetically engineered and pharmacologically treated cells and mice were used to determine whether NR1D1 modulates calcium homeostasis in the SR. The translational impact of our finding was then tested in human muscle cells obtained from patients with DMD and in a mouse model for DMD. Based on age and weight, animals were assigned to the different experimental groups. The number of samples for in vivo and in vitro assays was based on our experience and publications in the field (14, 21, 29).

### Cell culture and treatments

#### Mouse and human muscle cells.

C2C12 cells (ATCC) were cultured in high-glucose DMEM (41965039, Gibco, Thermo Fischer Scientific) supplemented with 10% FBS and 0.4% gentamycin and differentiated by replacing the previous medium with DMEM supplemented with 2% horse serum and 0.4% gentamycin for 5 days. Primary myoblasts from controls and patients with DMD, provided by Myobank-AFM (Myology Institute, Pitié-Salpêtrière Hospital, Paris, France), were cultured in DMEM supplemented with 20% fetal calf serum and 0.2% primocin. Immortalized myoblasts from healthy controls and patients with DMD, provided by MyoLine (Myology Institute), were cultured in M199/DMEM (1V/3V) supplemented with 20% FBS, 25 μg/mL fetuin, 0.5 ng/mL bFGF, 5 ng/mL EGF, 5 μg/mL insulin, 0.2 μg/mL dexamethasone, and 50 μg/mL gentamycin. Differentiation was initiated in DMEM containing 5 μg/mL insulin and 50 μg/mL gentamycin.

#### NR1D1 and Mln overexpression in C2C12 cells.

Generation of NR1D1- and MLN-overexpressing C2C12 cells was performed as previously described (5, 14). Briefly, mouse *Mln* and human *NR1D1* coding sequences were inserted into the pBabe plasmid (Addgene) by using BamHI-SalII restriction sites. NR1D1 and Mln or empty pBabe plasmids were transfected into Phoenix cells using JetPEI (Polyplus). Next, the supernatant of Phoenix cell culture was incubated with C2C12 cells, leading to their infection by retroviruses. The selection was done by a 15-day treatment with puromycin for NR1D1-overexpressing cells and neomycin for Mln-overexpressing cells.

#### Lentivirus-mediated overexpression of MLN in human myoblast cell lines.

cDNA encoding human *MLN* was amplified by RT-PCR and subcloned into the pLenti-IRES-BSD vector via EcoRI and XhoI restriction sites to produce the recombinant lentivirus plasmid pLenti-hMRLN-IRES-BSD. The day before the transfection, 2 × 10^6^ of 293T cells were plated in 10 cm culture dishes in DMEM proliferation medium supplemented with 10% FBS and 1% gentamycin. HEK293T cells were transfected using the jetPEI Transfection Reagent with 9 μg pLenti-IRES-BSD or plenti-hMRLN-IRES-BSD vector. In both cases, cells were cotransfected with 4.5 μg pMDL, 2.7 μg VSVG, and 1.8 μg pREV plasmids to permit the production of the viral particles. After 24 hours, media were replaced. Seventy-two hours later supernatants containing viruses were collected and filtered through a 0.45 μm filter. Human immortalized myoblasts were plated the day before infection and infected overnight at approximately 40% confluency with a media mixed 1:1 with viral supernatant (supplemented with Medium 199 [Gibco, Thermo Fischer Scientific], fetuin, bFGF, EGF, insulin, dexamethasone, and FBS), in presence of 4 μg/mL polybrene (MilliporeSigma). Twenty-four hours later, supernatant was removed and fresh medium was added. Infected myoblast cells were screened using 16 μg/mL blasticidin for 10 days. The remaining cells were evaluated for overexpression of human myoregulin by qPCR.

#### Lentiviral knockdown of MLN in human DMD myoblast cell lines.

Stable *MLN* silencing in immortalized DMD human myoblasts was achieved using a Dharmacon SMARTvector lentiviral shRNA delivery system according to the manufacturer’s instructions. Briefly, cells were infected, in the presence of 4 μg/mL polybrene, with virus expressing a nontargeting control or *MLN* shRNA (MOI 50). The percentage of RFP-positive cells was checked 48 hours after infection. SMARTvector Lentiviral Particles (catalog no. V3SH7945-245505968 and V3SH7945-245224017; Dharmacon, Horizon Discovery Ltd.) toward *MLN* targeted ACCACTACCTGGGATTAAT and GGACTTCGCTTATTGAACC sequences, respectively. NonTargeting SMARTvector shRNA Lentiviral particles (catalog no. S-005000) were used as an infection control

#### Pharmacological modulation of NR1D1 activity.

Pharmacological modulation of NR1D1 was obtained by adding in the culture medium either the synthetic agonist SR9009 (10 μM) or the synthetic antagonist SR8278 (10 μM), both from Merck Chimie SAS. TG was dissolved in DMSO, which was added at the same concentration in control conditions.

### Mice housing and treatments

All mice were housed in our animal facility with a 12-hour light/12-hour dark cycle and had free access to food and water. *Nr1d1*-deficient (*Nr1d1^–/–^*) mice and skeletal muscle–specific NR1D1 DBD mutant (*Nr1d1 DBDmut^fl/fl^*;*MCK^Cre/+^*) mice expressing a truncated NR1D1 lacking the DBD were generated as previously described (14, 30) and compared with respective control littermates.

Gastrocnemius muscles were collected at 2 pm and were either flash-frozen in liquid nitrogen or rapidly frozen using isopentane cooled with liquid nitrogen for immunostaining or freshly processed for microsome preparation. The effect of pharmacological NR1D1 activation on SR calcium uptake was tested in gastrocnemius muscle collected from wild-type mice treated with SR9009 (100 mg/kg) or its vehicle twice daily for 3 days (21).

To evaluate the therapeutic potential of NR1D1 activation in DMD, 25-week-old *mdx/Utr^+/–^* (31) mice were treated with SR9009 (100 mg/kg, once a day for 20 days) or vehicle.

### Creatine phosphokinase activity

Blood creatine phosphokinase activity was measured with the creatine kinase assay kit (ab155901, Abcam), according to the manufacturer’s instructions.

### Muscular hydroxyproline assay

4-hydroxyproline, a major component of collagen, was detected using the MAK008 assay kit (MilliporeSigma), according to the manufacturer’s instructions. Briefly, muscle (10 mg) was homogenized in 100 μL water, and hydrolysis was started by adding 100 μL 12 M HCl. After 3 hours at 120°C, samples were spun down at 10,000*g*. 20 μL of the resulting supernatant was transferred in a 96-well plate and evaporated under vacuum. 100 μL chloramine T/oxidation buffer mixture was added into the wells. Then, 100 μL DMAB reagent was added. The plate was incubated for 90 minutes at 60°C. Absorbance was measured at 560 nm and compared with hydroxyproline standards.

### In situ contractile properties of the gastrocnemius muscle

Mice were deeply anesthetized with intraperitoneal injections of ketamine (50 mg/kg) and dexmedetomidine (Domitor, 0.25 mg/kg). The dissection protocol was as previously described (32). Briefly, all the muscles of the right hind limb were denervated, except the gastrocnemius muscle, which was isolated from surrounding tissues. Then, the limb was immersed in a thermostatically controlled (37°C) bath of paraffin oil and fixed with bars and pins. The gastrocnemius muscle was maintained in a horizontal position, and its distal tendon was connected to a force transducer (Grass FT 10, Grass Instruments). The muscle length was adjusted to produce a maximal twitch peak tension. Contractions were induced by stimulation of the sciatic nerve (0.2 ms pulses) through bipolar platinum electrodes at twice the minimum voltage required to obtain the maximal twitch response. At the end of the recording session, the muscle was removed for determination of muscle wet weight, frozen in liquid nitrogen, and stored at −80°C.

### RT-qPCR analysis

RNAs were extracted from mouse muscle, C2C12 cells, and human myotubes seeded in 6-well plates or from gastrocnemius muscle, according to the Trizol (Invitrogen, Thermo Fischer Scientific)/chloroform/isopropanol protocol. After DNase treatment, cDNA was obtained using the High-Capacity cDNA Reverse Transcription Kit (Life Technologies). qPCRs were realized using the SYBR Green Real-Time PCR Master Mix kit (Agilent Technologies) and a MX3005 apparatus (Agilent Technologies). Mouse- and human-specific primers are provided in Supplemental Tables 1 and 2, respectively. Gene expression was normalized to cyclophilin A (*Ppia*).

### SERCA-dependent Ca2+ uptake

Gastrocnemius muscles were collected and homogenized at 4°C in a dedicated buffer (Tris-HCl, pH = 7, 1 M, 8% sucrose, 1 mM PMSF, 2 mM DTT) with a Polytron (Kinematica AG). Samples were then centrifuged at 1,300*g* at 4°C for 10 minutes in order to remove nuclei. The supernatant obtained after a second centrifugation (20,000*g*, 4°C, 20 min) corresponds to the enriched microsomal fraction. 150 μg proteins were placed in calcium uptake buffer (120 μM CaCl_2_, 150 μM EGTA, 30 mM Tris-HCl, pH= 7, 100 mM KCl, 5 mM NaN_3_, 6 mM MgCl_2_, 10 mM oxalate) and put in 2 mL Oxygraph-2k chambers (Oroboros Instruments) equipped with the fluorescence LED2 module. Calcium green probe (1 μM) and ATP (5 mM) were added, and fluorescence was measured over time (λex 506 nm, λem 531 nm). Ca^2+^ (10 μM) pulse was then injected into the chambers. Finally, TG (1 μM) was added in order to ensure that SERCA-dependent Ca^2+^ uptake was measured. To reflect SERCA-dependent Ca^2+^ uptake, we calculated the slope of the fluorescence intensity decrease subtracted by the residual slope measured in presence of TG.

### SR Ca2+ content in C2C12 cells

Experiments were conducted following a technical protocol adapted from Ducastel et al. (33). C2C12 cells and human myoblasts were plated in a 96-well plate (20,000/well) and differentiated for 4 days. Then, medium was replaced for 24 hours by low Ca^2+^ concentration Locke’s buffer (154 mM NaCl, 4 mM NaHCO_3_, 5 mM KCl, 0.1 mM CaCl_2_ 2H_2_O, 1 mM MgCl_2_ 6H_2_O, 5 mM glucose, 10 mM HEPES, pH = 7.4), as previously described (34). To detect cytosolic Ca^2+^, myotubes were then loaded with Fluo4-AM (λex 490 nm, λem 516 nm) for 30 minutes at 37°C, with 5% CO_2_ in free Ca^2+^ Locke’s buffer. Following 2 washes with 2.3 mM Ca^2+^ Locke’s buffer, TG (1 μM) was added in order to deplete SR Ca^2+^ store. Fluorescence intensity was immediately recorded every 10 seconds over 5 minutes using a microplate reader (Infinite 200 pro, Tecan) in order to estimate SR Ca^2+^ content until stabilization.

### Calcium imaging

Cells were grown on glass-bottom dishes for calcium-imaging experiments. Ratiometric dye Fura-2/AM (F1221, Invitrogen, Thermo Fischer Scientific) was used as a Ca^2+^ indicator. Cells were loaded with 2 μM Fura-2/AM for 45 minutes at 37°C and 5% CO_2_ in corresponding medium and subsequently washed 3 times with external solution containing 140 mM NaCl, 5 mM KCl, 1 mM MgCl_2_, 2 mM CaCl_2_, 5 mM glucose, 10 mM HEPES (pH = 7.4). The glass-bottom dish was then transferred in a perfusion chamber on the stage of Nikon Eclipse Ti microscope. Fluorescence was alternatively excited at 340 and 380 nm with a monochromator (Polychrome IV, TILL Photonics GmbH) and captured at 510 nm by a QImaging CCD camera (QImaging, Teledyne Photometrics). Acquisition and analysis were performed with MetaFluor 7.7.5.0 software (Molecular Devices Corp.).

### Tissue histology

Cross-sectional area was analyzed as previously described (21, 35). Conventional H&E staining was performed to describe histological status of muscle sections (36). Sirius red staining was performed to describe fibrosis (29).

### ChIP experiment

ChIP assays were performed as previously described (37) with minor modifications as follows. Gastrocnemius muscles from wild-type C57/Bl6 mice were homogenized in LB1 buffer (10 mM HEPES-KOH, pH = 7.5, 0.5% NP-40, 5 mM MgCl_2_, 500 μM DTT, 3 μg/mL Cytochalasin B (MilliporeSigma), protease inhibitor cocktail) and cross-linked with 1% paraformaldehyde for 10 minutes at room temperature. Chromatin was sheared over 90 minutes using the Bioruptor (Diagenode) coupled to a water-cooling system and subsequently concentrated with a centricon 10 kDa column (MilliporeSigma). 50 μg chromatin was immunoprecipitated overnight at 4°C with an antibody against NR1D1 (13418S, ChIP grade, Cell Signaling Technology). BSA/yeast tRNA-blocked Protein A/G dynabeads (Invitrogen, Thermo Fischer Scientific) were then added for 6 hours at 4°C while agitating and washed. Cross-linking was reversed by incubating precipitated chromatin overnight at 65°C. DNA was purified using the QIAquick PCR purification kit (Qiagen) and was analyzed by qPCR using Brilliant II SYBR Green QPCR Master Mix (Agilent Technologies) and specific primers (Supplemental Table 3).

### Data availability

Publicly available GSE data were analyzed with the GEO2R tool available on the NCBI website (https://www.ncbi.nlm.nih.gov/geo/geo2r/) (GEO GSE6011, GSE3307, and GSE109178). Benjamini and Hochberg (false discovery rate) was applied to the *P* values. Data were then analyzed on GraphPad Prism 9.0.

### Statistics

The number of biological replicates, *n*, which was chosen on the basis of our experience and good laboratory practice, is reported in each figure legend. Data are shown as the mean ± SEM. Analysis was performed with GraphPad Prism software 9.0. One-way ANOVA followed by Tukey’s post-hoc tests were carried out in order to establish statistical significance when comparing 3 groups or more. Unpaired or paired 2-tailed Student’s *t* tests were used to compare 2 groups, as indicated in figure legends. *P* values of less than 0.05 were considered significant.

### Study approval

All the described animal procedures were approved by the local ethics committee (Animal Experimentation Ethics Committee [Comité d’Ethique en Expérimentation Animale], Lille, France; CEEA75).

## Author contributions

AB, CD, and SL conceived and designed the experiments. AB, CD, AML, BP, YS, KK, AH, MG, CG, VM, SD, MC, QT, MZ, JB, AF, JO, and LF acquired and analyzed results. AB, CD, AML, BP, YS, KK, NP, FPR, BB, HD, and SL interpreted data. AB, HD, and SL wrote the original draft manuscript. AB, CD, BP, AML, YS, KK, NP, FPR, BB, BS, HD, and SL reviewed and edited the manuscript. All authors approved the final version. AB and CD share first authorship. AB is listed first in the order because he initiated the project and contributed to the original draft.

## Supplementary Material

Supplemental data

## Version 1 08/02/2022

In-Press Preview

## Version 2 09/08/2022

Electronic publication
