# Supplementary material for: NR1D1 controls skeletal muscle calcium homeostasis through myoregulin repression
Source: JCI Insight. 2022 Sep 8;7(17):e153584. doi: 10.1172/jci.insight.153584 (PMC9536258; doi:10.1172/jci.insight.153584)
Supplement: Supplemental data [file jciinsight-7-153584-s188.pdf]

Supplemental Figure S1

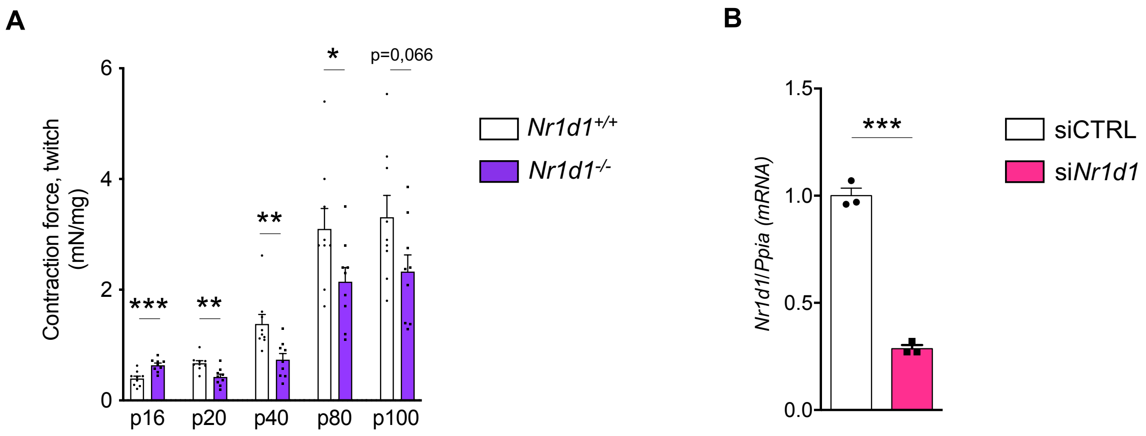

Supplemental Figure S1. *NR1D1* modulates mouse muscle contraction. (**A**) Tetanic contraction of *tibialis anterior* muscle obtained with incremental frequency stimulation (from 16Hz to 100Hz) in *Nr1d1*$^{+/+}$ and *Nr1d1*$^{-/-}$ mice (n=9). * p<0,05, ** p<0,01 and *** p<0,001. (**B**) *Nr1d1* expression in C2C12 transfected with si*Nr1d1* (n=3).

Supplemental Figure S2

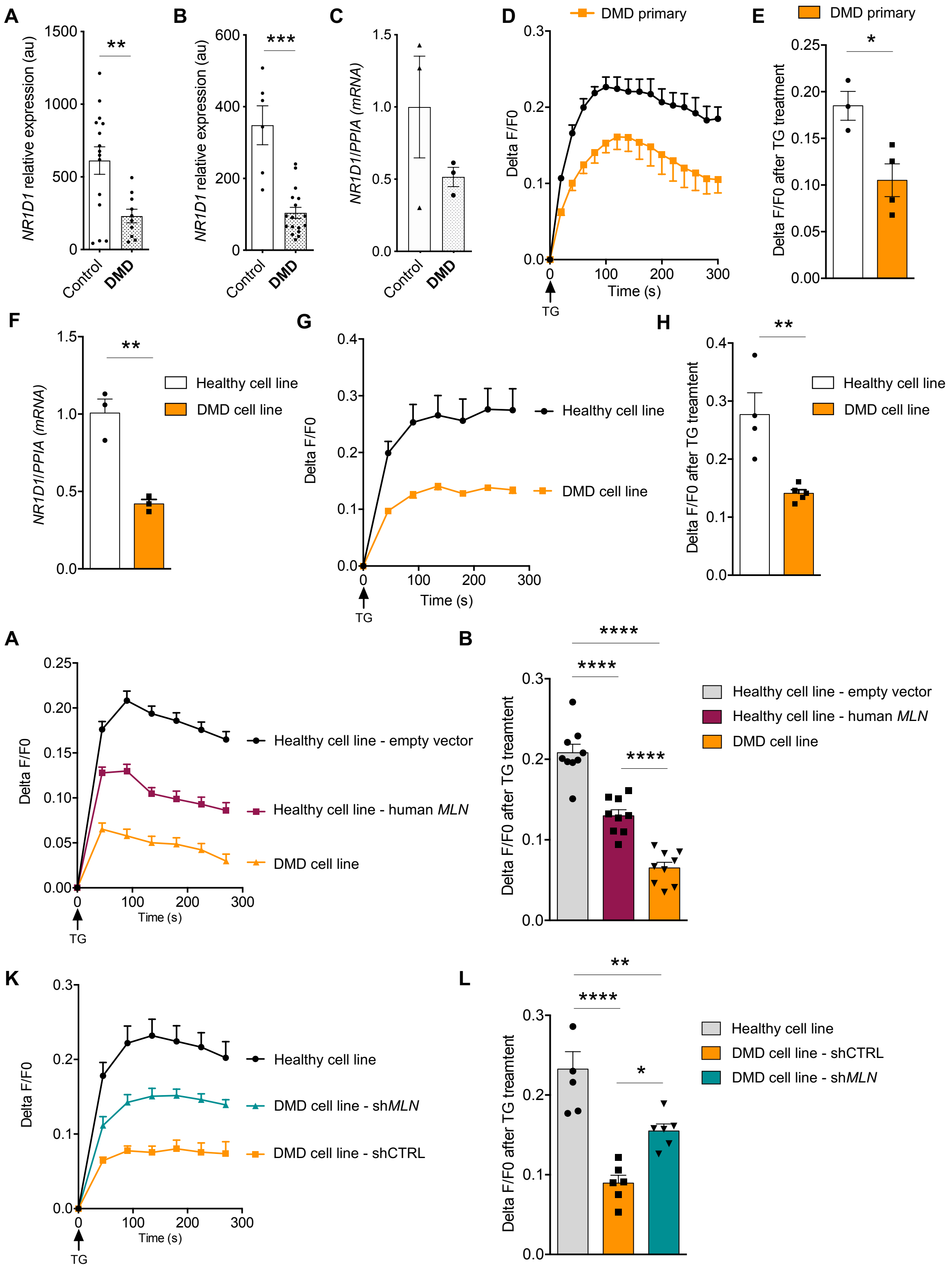

Supplemental Figure S2. *MLN* downregulation in myotubes from patients suffering from Duchenne muscular dystrophy (DMD) restores ER calcium content. Data from (**A**) GSE3307

probe 204769, (**B**) GSE109178 probe 31637. **p<0.01, ***p<0.001 *vs*. control, unpaired t-test. (**C**) RTqPCR results obtained in dorsal muscles from control and DMD patients provided by the French Myobank, n=3 independent samples in each group. (**D**) Representative curves and (**E**) peak fluorescence intensity of thapsigargin (TG)-induced Sarcoplasmic Reticulum (SR) Ca^{2+} release in primary myotubes from healthy controls or patients suffering from Duchenne Muscular Dystrophy (DMD). Cells are loaded with Fluo4-AM and SR Ca^{2+} release is induced by the addition of 1µM TG. Results are expressed as means ± SEM of the Delta F/F0 ratio, n=3 healthy controls, n=4 in DMD groups. *p=0.0110 *vs.* control cells, unpaired t-test. (**F**) Representative curves and (**G**) peak fluorescence intensity of TG-induced SR Ca^{2+} release in immortalized myotubes from healthy controls or patients suffering from DMD. Cells are loaded with Fluo4-AM and SR Ca^{2+} release is induced by the addition of 1 µM TG. Results are expressed as means ± SEM of the Delta F/F0 ratio, n=4 healthy controls, n=5 in DMD groups. **p=0.005 *vs.* control cells, unpaired t-test. (**H**) *NR1D1* expression in healthy control or DMD immortalized myotubes, n=5-7. *p=0.0035 *vs.* control cells in panel B, unpaired t-test. (**I**) Representative curves and (**J**) peak fluorescence intensity of TG-induced Sarcoplasmic Reticulum (SR) Ca^{2+} release in immortalized myotubes from healthy controls or patients suffering from DMD with overexpression of the human MLN or the corresponding empty vector. Cells are loaded with Fluo4-AM and SR Ca^{2+} release is induced by the addition of 1µM TG. Results are expressed as means ± SEM of the Delta F/F0 ratio, n=9 replicates for each group. ****p<0.0001 healthy control + human MLN *vs.* healthy control + empty vector, ****p<0.0001 healthy control + human MLN *vs.* DMD, ****p<0.0001 healthy control + empty vector *vs.* DMD 1-way ANOVA, Tukey's multiple comparison test. (**K**) Representative curves and (**L**) peak fluorescence intensity of TG-induced Sarcoplasmic Reticulum (SR) Ca^{2+} release in immortalized myotubes from healthy controls or patients suffering from DMD with shCTRL or shMLN. Cells are loaded with Fluo4-AM and SR Ca^{2+} release is induced by the

addition of 1 μM TG. Results are expressed as means ± SEM of the Delta F/F0 ratio, n=9 replicates for each group. ****$p<0.0001$ healthy control *vs.* DMD + shCTRL, **$p<0.0055$ healthy control *vs.* DMD + shMLN, *$p<0.0174$ DMD + shCTRL *vs.* DMD + shMLN, 1-way ANOVA, Tukey's multiple comparison test.

Supplemental Figure S3

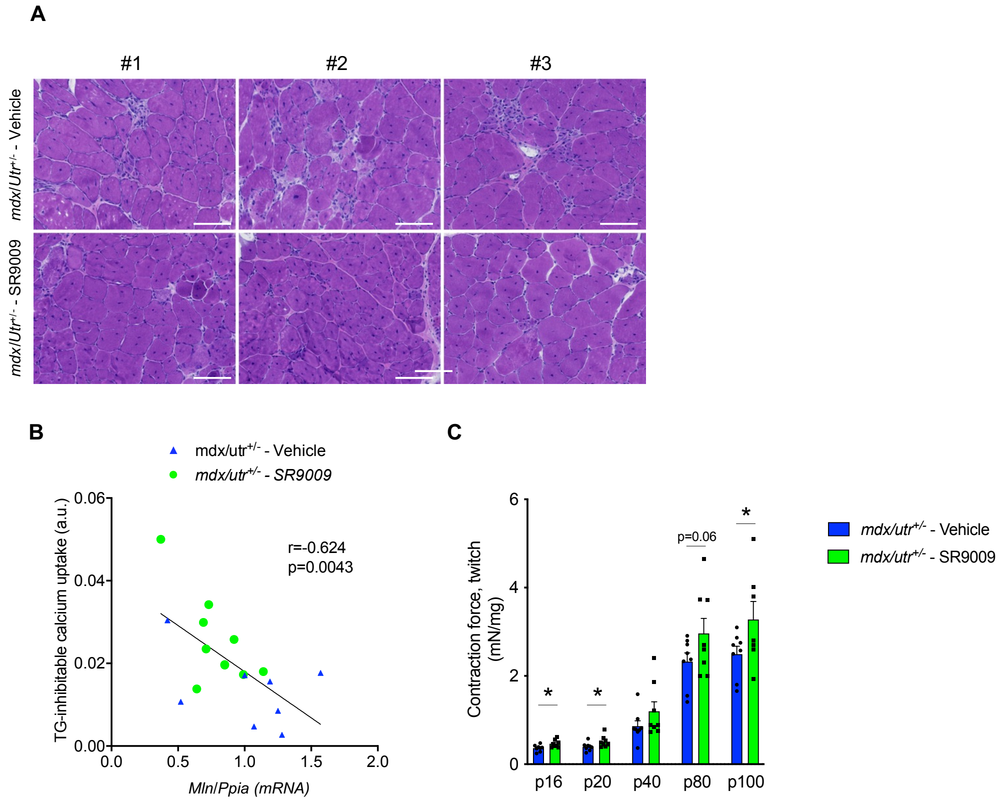

Supplemental Figure 3. Effects of SR9009 on muscles from *mdx/Utr*$^{+/-}$ mice. (**A**) H&E staining on *tibialis anterior* sections from three different (#1, #2, #3) *mdx/Utr*$^{+/-}$ mice treated with SR9009 or vehicle for 20 days. Scale bars indicate 100µm. (**B**) Pearson correlation analysis between *Mln* expression and SERCA activity in muscle from vehicle- or SR9009-treated *mdx/Utr*$^{+/-}$ mice. (**C**) Tetanic contraction of *tibialis anterior* muscle obtained with incremental frequency stimulation (from 16Hz to 100Hz) in vehicle- and SR9009-injected *mdx/Utr*$^{+/-}$ mice, n=8-9, * $p<0,05$.

Supplemental Table S1: Mouse RTqPCR primers

target	Accession number	forward 5'-3'	reverse 3'-5'
Serca1	NM_007504	AAGGAGCCCAGATCAACAGGCA	ACTCCCACAGAGACTTGCCTTCCTC
Serca2	NM_009722	GCCATCAGCCAAGTCTCCACAT	AGCTGGCTGCACACCTAAACAA
Ryr1	NM_009109	ACGTACAGTCAGGTGGCTCAGA	CCAGCACAATGAGGTCCTGGTC
Mln	NM_001304739	GTTGCACCCCTGAACAGAACCA	CCTCAGGAGGTAGCAGGTAGCA
Pdgfra	NM_001083316.2	TGCTAGCGCGGAACCTCAGA	ATAGCTCCTGAGACCCGCTG
Col1a2	NM_007743.2	CTGCTGGTGTTCGTGGTTC	CGGCTGTATGAGTTCTTC
Ppia	NM_008907	GCATACGGGTCCTGGCATCTTGTCC	ATGGTGATCTTCTTGCTGGTCTTGC

Supplemental Table S2: Human RTqPCR primers

target	Accession number	forward 5'-3'	reverse 3'-5'
MLN	NM_001304732	TCCCTTGACTTTGGACTTCGCT	TCAGCACAGGTGGTCTCTTAGC
NR1D1	NM_021724	ATCAATCGCAACCGCTGCCAGC	TTGGGGATGCGCCCAAAACGC
PPIA	NM_001123068	GCATACGGGTCCTGGCATCTTGTCC	ATGGTGATCTTCTTGCTGGTCTTGC

Supplemental Table S3: ChIP qPCR primers

**target**	**forward 5'-3'**	**reverse 3'-5'**
Site -1.4kb	TATCTGATACGCAGGTTATCTG	GGGAGAGGGTGTGCAAGTTA
Site -5.4kb	GGCCAGATCTGCTTTAGTATG	CAGGGTGGCTACATTACTCA
Site -6.7kb	GCAGGACATCTCTGACACC	TCAGAGTTCTCTGGCTTTCAG
negative control region	CTGCAGCCCCTTCAGAGGT	CAACCTTGCTAGTGCTAAAAC
